# Bis[(diamino­methyl­idene)aza­nium] 5-(1-oxido-1*H*-1,2,3,4-tetra­zol-5-yl)-1*H*-1,2,3,4-tetra­zol-1-olate

**DOI:** 10.1107/S1600536812014456

**Published:** 2012-04-13

**Authors:** Rong Fan, Ping Li, Seik Weng Ng

**Affiliations:** a Shanghai Institute of Materia Medica, Chinese Academy of Sciences, Shanghai 201203, People’s Republic of China; b Jining Teachers College, Department of Chemistry, Wulanchabu 012000, Inner Mongolia, People’s Republic of China; c Department of Chemistry, University of Malaya, 50603 Kuala Lumpur, Malaysia; d Chemistry Department, King Abdulaziz University, PO Box 80203 Jeddah, Saudi Arabia

## Abstract

The anion of the title salt, 2[C(NH_2_)_3_]^+^·C_2_N_8_O_2_^2−^, lies on a center of inversion and its two five-membered rings are coplanar. The guanidinium cation forms N—H⋯O and N—H⋯N hydrogen bonds to the anion, generating an eight-membered ring. Other hydrogen bonds lead to the formation of a three-dimensional network.

## Related literature

For the synthesis of 1,1′-dihy­droxy-5,5′-bis­tetra­zole, see: Tselinskii *et al.* (2001 ▶). 
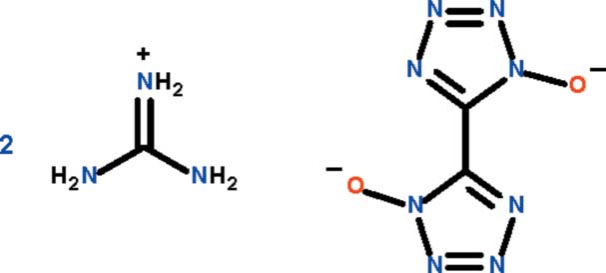


## Experimental

### 

#### Crystal data

2CH_6_N_3_^+^·C_2_N_8_O_2_^2−^
*M* *_r_* = 288.28
Monoclinic, 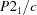

*a* = 3.6477 (3) Å
*b* = 16.9661 (12) Å
*c* = 9.5465 (7) Å
β = 97.465 (1)°
*V* = 585.80 (8) Å^3^
*Z* = 2
Mo *K*α radiation
μ = 0.13 mm^−1^
*T* = 293 K
0.12 × 0.11 × 0.08 mm

#### Data collection

Bruker SMART APEX diffractometer
3402 measured reflections
1328 independent reflections
1229 reflections with *I* > 2σ(*I*)
*R*_int_ = 0.021

#### Refinement

*R*[*F* ^2^ > 2σ(*F* ^2^)] = 0.039
*wR*(*F* ^2^) = 0.107
*S* = 1.02
1328 reflections
115 parameters
6 restraints
All H-atom parameters refined
Δρ_max_ = 0.24 e Å^−3^
Δρ_min_ = −0.25 e Å^−3^

### 

Data collection: *APEX2* (Bruker, 2009 ▶); cell refinement: *SAINT* (Bruker, 2009 ▶); data reduction: *SAINT*; program(s) used to solve structure: *SHELXS97* (Sheldrick, 2008 ▶); program(s) used to refine structure: *SHELXL97* (Sheldrick, 2008 ▶); molecular graphics: *X-SEED* (Barbour, 2001 ▶); software used to prepare material for publication: *publCIF* (Westrip, 2010 ▶).

## Supplementary Material

Crystal structure: contains datablock(s) global, I. DOI: 10.1107/S1600536812014456/bt5870sup1.cif

Structure factors: contains datablock(s) I. DOI: 10.1107/S1600536812014456/bt5870Isup2.hkl

Supplementary material file. DOI: 10.1107/S1600536812014456/bt5870Isup3.cml

Additional supplementary materials:  crystallographic information; 3D view; checkCIF report

## Figures and Tables

**Table 1:** Hydrogen-bond geometry (Å, °)

*D*—H⋯*A*	*D*—H	H⋯*A*	*D*⋯*A*	*D*—H⋯*A*
N5—H1⋯O1	0.89 (1)	1.94 (1)	2.821 (2)	173 (2)
N5—H2⋯N3^i^	0.88 (1)	2.40 (1)	3.196 (2)	152 (2)
N6—H3⋯N3^i^	0.88 (1)	2.34 (1)	3.126 (2)	149 (2)
N6—H4⋯N4^ii^	0.88 (1)	2.12 (1)	2.975 (2)	164 (2)
N7—H5⋯O1^iii^	0.87 (1)	1.97 (1)	2.754 (2)	150 (2)
N7—H6⋯N2	0.88 (1)	2.23 (1)	3.104 (2)	177 (2)

Symmetry codes: (i) 
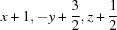
; (ii) 
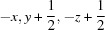
; (iii) 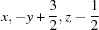.

**Fig. 1.**
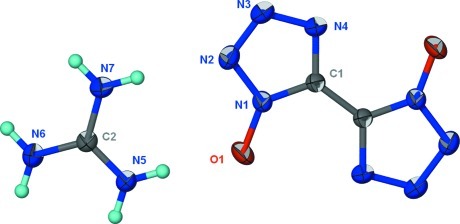
Anisotropic displacement ellipsoid plot (Barbour, 2001) of 2[C(NH2)3]+ (C2N8O2)2- at the 50% probability level; hydrogen atoms are drawn as spheres of arbitrary radius. The dianion lies on a center of inversion; symmetry-related atoms are not labeled.

## supplementary crystallographic information

### Comment

The depronated ion of 1,1'-dihydroxy-5,5'-bistetrazole is an example of an organic compound having no hydrogen atoms, and is an appropriate building block for the synthesis of coordination polymers that require metal–nitrogen linkages. To date, no crystal structure of the metal derivatives have been reported. The ion is obtained by the reaction of 1,1'-dihydroxy-5,5'-bistetrazole with guanidine. In the salt (Scheme I), the anion lies on a center of inversion; its two five-membered rings are necessarily coplanar. The guanidinium cation forms N–H···O and N–H···N hydrogen bonds to the anion to generate an eight-membered ring (Fig. 1). Other hydrogen bonds lead to the formation of a three-dimensional network (Table 1).

### Experimental

Guanidine carbonate (180 mg, 1 mmol) was added to a methanol solution (10 ml) of 1,1'-dihydroxy-5,5'-bistetrazole (206 mg, 1 mmol). The mixture was stirred for 2 h. The white precipitate that formed was filtered and washed with methanol; yield 0.245 g (90%). CH&N Elemental analysis for C_4_H_8_N_14_O_2_: Calc. C 16.90, H 2.84, N 69.00%. Found C 16.74, H 2.87, N 68.23%. Diethyl ether was used to recrystallize the compound.

### Refinement

The amino H-atoms were located in a difference Fourier map, and were refined with a distance restraint of N–H 0.88±0.01 Å; their displacement parameters were refined.

### Crystal data

**Table:**

2CH_6_N_3_^+^·C_2_N_8_O_2_^2^^−^	*F*(000) = 300
*M**_r_* = 288.28	*D*_x_ = 1.634 Mg m^−^^3^
Monoclinic, *P*2_1_/*c*	Mo *K*α radiation, λ = 0.71073 Å
Hall symbol: -P 2ybc	Cell parameters from 2581 reflections
*a* = 3.6477 (3) Å	θ = 2.5–28.5°
*b* = 16.9661 (12) Å	µ = 0.13 mm^−^^1^
*c* = 9.5465 (7) Å	*T* = 293 K
β = 97.465 (1)°	Prism, colorless
*V* = 585.80 (8) Å^3^	0.12 × 0.11 × 0.08 mm
*Z* = 2	

### Data collection

**Table:**

Bruker SMART APEX diffractometer	1229 reflections with *I* > 2σ(*I*)
Radiation source: fine-focus sealed tube	*R*_int_ = 0.021
Graphite monochromator	θ_max_ = 27.5°, θ_min_ = 2.5°
ω scans	*h* = −4→4
3402 measured reflections	*k* = −20→21
1328 independent reflections	*l* = −7→12

### Refinement

**Table:**

Refinement on *F*^2^	Primary atom site location: structure-invariant direct methods
Least-squares matrix: full	Secondary atom site location: difference Fourier map
*R*[*F*^2^ > 2σ(*F*^2^)] = 0.039	Hydrogen site location: inferred from neighbouring sites
*wR*(*F*^2^) = 0.107	All H-atom parameters refined
*S* = 1.02	*w* = 1/[σ^2^(*F*_o_^2^) + (0.0648*P*)^2^ + 0.1424*P*] where *P* = (*F*_o_^2^ + 2*F*_c_^2^)/3
1328 reflections	(Δ/σ)_max_ = 0.001
115 parameters	Δρ_max_ = 0.24 e Å^−^^3^
6 restraints	Δρ_min_ = −0.25 e Å^−^^3^

### Fractional atomic coordinates and isotropic or equivalent isotropic displacement parameters (Å^2^)

**Table:**

	*x*	*y*	*z*	*U*_iso_*/*U*_eq_	
O1	0.3597 (3)	0.63225 (6)	0.54252 (10)	0.0478 (3)	
N1	0.1684 (3)	0.59470 (6)	0.43882 (10)	0.0326 (3)	
N2	0.1200 (3)	0.62317 (6)	0.30734 (11)	0.0409 (3)	
N3	−0.0687 (3)	0.56977 (7)	0.22907 (12)	0.0431 (3)	
N4	−0.1457 (3)	0.50828 (6)	0.30661 (11)	0.0377 (3)	
N5	0.5844 (4)	0.79085 (7)	0.52906 (12)	0.0447 (3)	
N6	0.5689 (4)	0.90966 (7)	0.41611 (13)	0.0441 (3)	
N7	0.2983 (4)	0.80194 (7)	0.30217 (12)	0.0427 (3)	
C1	0.0040 (3)	0.52416 (6)	0.43820 (11)	0.0283 (3)	
C2	0.4842 (3)	0.83418 (7)	0.41568 (12)	0.0324 (3)	
H1	0.525 (5)	0.7400 (6)	0.528 (2)	0.056 (5)*	
H2	0.703 (5)	0.8143 (11)	0.6033 (15)	0.064 (5)*	
H3	0.699 (5)	0.9327 (11)	0.4888 (16)	0.065 (5)*	
H4	0.485 (5)	0.9392 (9)	0.3427 (15)	0.061 (5)*	
H5	0.242 (5)	0.8300 (10)	0.2262 (15)	0.061 (5)*	
H6	0.248 (5)	0.7514 (6)	0.3000 (19)	0.057 (5)*	

### Atomic displacement parameters (Å^2^)

**Table:**

	*U*^11^	*U*^22^	*U*^33^	*U*^12^	*U*^13^	*U*^23^
O1	0.0689 (7)	0.0371 (5)	0.0365 (5)	−0.0201 (4)	0.0028 (4)	−0.0089 (4)
N1	0.0422 (5)	0.0260 (5)	0.0292 (5)	−0.0022 (4)	0.0034 (4)	−0.0017 (4)
N2	0.0550 (7)	0.0330 (5)	0.0343 (6)	−0.0011 (4)	0.0043 (5)	0.0064 (4)
N3	0.0527 (7)	0.0423 (6)	0.0321 (6)	−0.0019 (5)	−0.0028 (5)	0.0072 (4)
N4	0.0456 (6)	0.0365 (5)	0.0285 (5)	−0.0041 (4)	−0.0049 (4)	0.0021 (4)
N5	0.0657 (8)	0.0332 (6)	0.0318 (6)	−0.0096 (5)	−0.0073 (5)	0.0051 (4)
N6	0.0572 (7)	0.0303 (5)	0.0422 (6)	−0.0014 (5)	−0.0037 (5)	0.0048 (4)
N7	0.0584 (7)	0.0391 (6)	0.0287 (5)	−0.0054 (5)	−0.0021 (5)	0.0004 (4)
C1	0.0324 (5)	0.0244 (5)	0.0270 (5)	0.0011 (4)	−0.0004 (4)	−0.0007 (4)
C2	0.0360 (6)	0.0315 (5)	0.0294 (6)	−0.0006 (4)	0.0032 (4)	0.0016 (4)

### Geometric parameters (Å, º)

**Table:**

O1—N1	1.3013 (13)	N5—H2	0.877 (9)
N1—N2	1.3350 (14)	N6—C2	1.3172 (16)
N1—C1	1.3383 (14)	N6—H3	0.879 (10)
N2—N3	1.3112 (16)	N6—H4	0.883 (9)
N3—N4	1.3301 (15)	N7—C2	1.3200 (16)
N4—C1	1.3305 (14)	N7—H5	0.869 (9)
N5—C2	1.3201 (16)	N7—H6	0.876 (9)
N5—H1	0.889 (9)	C1—C1^i^	1.440 (2)
			
O1—N1—N2	122.03 (10)	H3—N6—H4	117.9 (18)
O1—N1—C1	129.56 (10)	C2—N7—H5	119.9 (13)
N2—N1—C1	108.36 (10)	C2—N7—H6	120.5 (12)
N3—N2—N1	106.34 (10)	H5—N7—H6	119.3 (18)
N2—N3—N4	110.97 (10)	N4—C1—N1	108.29 (10)
N3—N4—C1	106.04 (10)	N4—C1—C1^i^	127.50 (13)
C2—N5—H1	119.3 (12)	N1—C1—C1^i^	124.20 (13)
C2—N5—H2	117.7 (13)	N6—C2—N7	120.04 (11)
H1—N5—H2	123.0 (18)	N6—C2—N5	119.94 (11)
C2—N6—H3	122.5 (14)	N7—C2—N5	120.01 (11)
C2—N6—H4	119.5 (12)		
			
O1—N1—N2—N3	177.18 (11)	N3—N4—C1—C1^i^	179.84 (15)
C1—N1—N2—N3	−0.51 (14)	O1—N1—C1—N4	−177.19 (12)
N1—N2—N3—N4	0.58 (15)	N2—N1—C1—N4	0.26 (14)
N2—N3—N4—C1	−0.42 (14)	O1—N1—C1—C1^i^	3.0 (2)
N3—N4—C1—N1	0.09 (13)	N2—N1—C1—C1^i^	−179.50 (13)

Symmetry code: (i) −*x*, −*y*+1, −*z*+1.

### Hydrogen-bond geometry (Å, º)

**Table:**

*D*—H···*A*	*D*—H	H···*A*	*D*···*A*	*D*—H···*A*
N5—H1···O1	0.89 (1)	1.94 (1)	2.821 (2)	173 (2)
N5—H2···N3^ii^	0.88 (1)	2.40 (1)	3.196 (2)	152 (2)
N6—H3···N3^ii^	0.88 (1)	2.34 (1)	3.126 (2)	149 (2)
N6—H4···N4^iii^	0.88 (1)	2.12 (1)	2.975 (2)	164 (2)
N7—H5···O1^iv^	0.87 (1)	1.97 (1)	2.754 (2)	150 (2)
N7—H6···N2	0.88 (1)	2.23 (1)	3.104 (2)	177 (2)

Symmetry codes: (ii) *x*+1, −*y*+3/2, *z*+1/2; (iii) −*x*, *y*+1/2, −*z*+1/2; (iv) *x*, −*y*+3/2, *z*−1/2.
